# Modern Medical Therapeutics

**Published:** 1872-03

**Authors:** 


*Modern Medical Therapeutics. A compendium of recent formulæ, and specific Therapeutical directions.* By GEO. H. NAPHEYS, A. M., M. D. Third edition, revised and improved. Philadelphia: S. W. Butler, M. D., 1871.

This is a convenient and valuable work on therapeutics, and cannot fail to be of use to the student and practitioner of medicine. It is largely composed of selections from experienced medical men in relation to therapeutics.

The anthor in the preface says; " I have given the most space to affections, in which treatment has been found to be of most avail. All previous collections of therapeutical facts have been arranged with reference to the articles of the Materia Medica. The nosological plan here adopted, is, I believe, the most convenient to the busy practitioner. It enables him to turn at once to the therapeusis of a disease."

Although somewhat novel, the plan of the author is well carried out and the result is a valuable work on therapeutics.

